# The Effects of live- in rehabilitation on ARV adherence, abstinence from drugs and lifestyle modification in people who inject drugs (PWID) Living with HIV – A clinic review

**DOI:** 10.12669/pjms.38.ICON-2022.5780

**Published:** 2022-01

**Authors:** Aneela Hussain, Anum Rahim, Anila Sheikh, Ahsun Jiwani

**Affiliations:** 1 Dr. Aneela Hussain, MBBS, FCPS (Internal Medicine), FCPS (Infectious Diseases), Infectious Diseases Department, Indus Hospital & Health Network; 2 Dr. Anum Rahim, MBBS, MSc Epidemiology and Biostatistics, Indus Hospital Research Center, Indus Hospital & Health Network; 3 Ms. Anila Sheikh, M.A International Relations, Infectious Diseases Department, Indus Hospital & Health Network; 4 Mr. Ahsun Jiwani, BScN, MSc Epidemiology and Biostatistics, Indus Hospital Research Center, Indus Hospital & Health Network

**Keywords:** HIV, Rehabilitation, Adherence

## Abstract

**Background & Objective:**

HIV/AIDS is mostly seen in people who inject recreational drugs (PWID). Adherence has to be optimum for its treatment to be effective. Compliance to HIV medication has been problematic in PWID making HIV control difficult. Many studies in the past have validated educational activities like rehabilitation programs beneficial in maintaining regularity in medication intake. This brought us to the question of looking at such programs and its effects on our population. This study was conducted to assess the impact of other perspectives of abstinence and adherence including family support and employment status on a person’s willingness for treatment continuation and avoidance of drugs.

**Methods:**

A retrospective chart review of 241 PWID was conducted to assess adherence to antiretroviral agents (ARVs) and abstinence from recreational drugs post visit to the rehabilitation center. Associations with family support, marital status, employment, income and back to work status were also assessed.

**Results:**

Adherence to ARVs had significant statistical association with marital status (p=0.025), starting work again (p=0.001), family support (p=0.009), employment status (p=0.009) and monthly income (p=0.025). While family support (p=0.033), employment status (p<0.0001), Going back to work (p<0.0001), mode of travel to Rehabilitation center (p<0.0001) and monthly income (p=0.004) were associated with abstinence from drugs. Duration of rehabilitation or age had no effect on adherence or abstinence in our patient population of PWID.

**Conclusion:**

Family and spousal support and employment promote optimal ARV compliance and should be encouraged when starting ARVs. Enrollment in a long-term complementing educational program would further enhance ARV intake and abstinence.

## INTRODUCTION

Human immunodeficiency virus (HIV) infection causes a decrease in immunity, and predisposes to superadded infections and long standing illness.^1^ A survey done in the end of 2016, revealed that a population of 36.7 million are living with HIV/AIDS and 2.1 million comprised of those younger than 15 years. Since June 2017, the number of HIV positive people accessing antiretroviral therapy (ART) globally rose up to 20.9 million from 15.8 million in June 2015, 7.5 million in 2010, and less than one million in 2000.^2^ In Pakistan as many as 133,000 people are infected with HIV.^3^ It is estimated that people who inject drugs (PWID) are 22 times more likely to acquire HIV than the rest of the population.^2^ There are approximately 11.8 million PWID worldwide, and 13.1% of them are thought to be living with HIV.^4^ In Pakistan, 105,000 people are drug injectors out of which 37.8% roughly are infected.^5^ A high level of adherence around >95% is required for antiretroviral therapy (ART) to be effective.^6^,^7^ Adherence is a patient’s ability to follow a treatment plan, take medications at prescribed times and frequencies, and follow restrictions regarding food and other medications.^8^ Rehabilitation is any service or activity that can address the health related challenges that people living with HIV (PLHIV) can face, it provides support to lead effective lives in communities. As per WHO, drug adherence counselling programs optimize treatment goals and outcomes,^9^ Psychoeducational interventions have been reported during the past year as promoting adherence to antiretroviral agents (ARV). However, a study showed that the effect of the educational intervention was not durable suggesting long-term or repetitive interventions to produce a lasting impact on adherence, lifestyle modification and recreational drug sobriety.^10^,^11^ In Pakistan a study also showed around 55% effects of rehabilitation on adherence in PWID.^12^

This study was conducted to assess the impact of other perspectives of abstinence and adherence including family support and employment status on a person’s willingness for treatment continuation and avoidance of drugs.

## METHODS

This study is a retrospective medical chart review conducted at the, HIV Clinic of IHHN, which is a tertiary care hospital in Karachi. The study population is a cohort of PWID living with HIV active or previous IV drug use who have been to a rehabilitation unit at the start of the treatment. After approval by the Institutional Review Board (IRD_IRB_2019_05_006), all patients fulfilling the inclusion criteria from 1st October 2016 till 30th September 2018 were included in the study and were followed for adherence to medicines and, abstinence from recreational drugs for almost 1-year post rehabilitation center visit. Demographic variables like marital status, age, mode of travel to the clinic, employment and monthly income were assessed along with abstinence and adherence variables. Adherence to ARV was checked by pill count of remaining ARV by a healthcare provider, pharmacy refills/ pick up and self-reporting by the patient or their caregivers. While, abstinence from recreational drugs was checked by inquiring about it from patients or their attendants. PLHIV (confirmed by WHO approved rapid tests) 15 years and above who were currently injecting drugs or had in the past were included in the study while those not willing to take ARV were excluded.

### Statistical Analysis:

For adherence and abstinence, age, duration of rehabilitation, duration of IV drug use and back to work were compared using Kruskal Wallis test at less than 3 months, 3 to 6 months, 7 to 10 months and greater than 10 months. For adherence, Pearson’s chi square test was applied for work status, Fisher exact for monthly income, financial status, mode of travel, marital status and gender. While likelihood ratios were reported for family support. However, for abstinence, back to work and mode of travel were assessed using Pearson’s chi square while gender and marital status were assessed with Fisher exact test. Lastly, likelihood ratios were reported for family support, financial status and monthly income. P value of less than equal to 0.05 was considered significant for all variables.

## RESULTS

The data of 241 individuals was included in the analysis. Majority of the patients were males 222 (92.1%). The median age of the study participants was 31 (27-36) years. Almost half of the patients were single 114 (47.7%), followed by married individuals 107 (44.8%). More than half of the study participants did not have any family support 122 (51%) during the period of undergoing therapy, followed by those who had support from their parents 43 (18%). In terms of mode of travelling to antiretroviral therapy center (ARTC), the travelling for almost half of the participants was facilitated by different voluntary NGOs, followed by those who came to the ARTC on their own 84 (35%). The majority of the participants 41 (58.5%) had a monthly income of 10,000-30,000 PKR, followed by those who had a monthly income of >60,000 PKR 54 (22.4%). The median duration spent in rehab was 45 (40-60) days. Similarly, the median duration of IV drug abuse was 1095 (365-2007.5) days.

### Adherence to ART:

The participants were divided in different groups according to the time they adhered to the ART (i.e., <3 months, 3-6 months, 7-10 months and more than 10 months). Kruskal Wallis test, Pearson chi-square/fisher exact test, and likelihood ratio test was applied as appropriate to assess significant association of the independent variables with the time adhered to ART. Table-I shows statistically significant associations between adherences to ART and marital status (p= 0.025), starting work again (p=0.001), mode of travel to ARTC (p= <0.0001), having family support during therapy (p=0.009), employment status of the study participants (p= 0.009) and monthly income (p= 0.025). Adherence to ART for longer time periods was found consistently higher among individuals who were either single or married than those who were either separated or divorced. Furthermore, the individuals who were provided the transportation facility by the NGOs were found to adhere to ART for longer duration. Moreover, long term adherence was found among those individuals who had a monthly income of 10,000 PKR-30,000PKR. Statistically significant associations were not found between adherence to ART and gender, age, duration in rehabilitation, duration of IV drug use, and going back to work.

**Table I:** Adherence to ART.

	<3 Months	3-6 Months - n(%)	7-10 Month - n(%)	>10 Months - n(%)	Total - n(%)	P value
**Duration in rehabilitation/ Days^**	40	45 (40-60)	60 (40-60)	45(40-60)	45(40-60)	0.739¥
**Duration of IV drug use/Days^**	1460(456.3-3467.5)	1460(365-1825)	1460(365-2920)	730(365-1835)	1095(365-2007.5)	0.063¥
**Age/ Years^**	32(26-42)	31(26-36)	30.5(26-37.5)	32(27-35)	31(27-36)	0.891¥
**Back to work^**	2	40(2-60)	60(40-60)	45(40-60)	45 (35-65)	0.14¥
**Gender**						
Male	10(83.3)a	90(91.8)a	33(91.7)a	89(93.7)a	222(92.1)	0.211€
Female	0a	2(2)a	0	4(4.2)a	6(2.5)	
Transgender/MSM	2(16.7)a	6(6.1)a,b	3(8.3)a,b	2(2.1)b	13(5.4)	
**Marital Status**						
Single	4(33.3)a	53(54.6)a	17(47.2)a	40(42.6)a	114(47.7)	0.025€
Divorced	0a,b,c	1(1)c	3(8.3)b	1(1.1)a,c	5(2.1)	
Married	6(50)a,b	35(36.1)b	16(44.4)a,b	50(53.2)a	107(44.8)	
Separated	2(16.7)a	8(8.2)a,b	0b	3(3.2)b	13(5.4)	
**Work status/Back to work**						
Yes	3(27.3)a	45(47.9)a	19(54.3)a	65(73)b	132(57.6)	
0.001Τ						
No	8(72.7)a	49(52.1)a	16(45.7)a	24(27)b	97(42.4)	
**Mode of travel to the ARTC**						
Self	2(18.2)a,b	27(27.6)	10(27.8)b	45(47.4)a	84(35)	<0.0001€
Family	5(45.5)a	4(4.1)b	1(2.8)b	11(11.6)b	21(8.8)	
NGO	4(36.4)a	67(68.4)b	25(69.4)b	39(41.1)a	135(56.3)	
**Family Support**						0.009 λ
Parents	0a	15(15.3)a	6(16.7)a	22(23.7)a	43(18)	0.009 λ
Siblings	5(41.7)a	13(13.3)b	5(13.9)b	8(8.6)b	31(13)	
Spouse	3(25)a	7(7.1)b	3(8.3)a,b	17(18.3)b	30(12.6)	
Other	1(8.3)a	4(4.1)a	1(2.8)a	7(7.5)a	13(5.4)	
No support	3(25)a	59(60.2)b	21(58.3)b,c	39(41.9)a,c	122(51)	
**Employment Status**						0.009€
Employed	5(41.7)a,b	50(51)b	18(50)b	66(69.5)a	139(57.7)	0.009€
Unemployed/Not supported lives with family	0a	7(7.1)a	4(11.1)a	3(3.2)a	14(5.8)	
Unemployed supported by family	7(58.3)a	24(24.5)b	11(30.6)a,b	22(23.2)b	64(26.6)	
Street Based	0a,b	17(17.3)b	3(8.3)a,b	4(4.2)a	24(10)	
**Social Status (monthly Income)**						
<10000	0a	11(11.2)a	4(11.1)a	11(11.6)a	26(10.8)	0.025€
10000-30000	8(66.7)a,b	47(48)b	24(66.7)a,b	62(65.3)a	141(58.5)	
30000-60000	0a	7(7.1)a	2(5.6)a	11(11.6)a	20(8.3)	
>60000	4(33.3)a	33(33.7)a	6(16.7)a,b	11(11.6)b	54(22.4)	

Τ Pearson’s chi square test, € Fisher Exact Test, λ Likelihood Ratio test, ¥ Kruskul Wallis Test, ^Median(IQR).

### Abstinence from Drugs:

For the analysis of second objective i.e. abstinence from drugs, the participants were divided in different groups according to the time they abstained from IV drugs (i.e. <3 months, 3-6 months, 7-10 months and more than 10 months). Kruskal Wallis test, Pearson chi-square/fisher exact test, and likelihood ratio test was applied as appropriate to assess significant association of the independent variables with the time the participants adhered from drugs. Table 2 shows significant associations between abstinence from IV and recreational drugs and having family support during therapy (p=0.03), Mode of travel to ARTC (p=<0.0001), employment status (p=<0.0001), and monthly income (p=0.004). Individuals who received transport to ARTC via NGOs were more likely to have longer duration of drug abstinence. Abstinence from drugs was found consistently higher among individuals who had family support. Similarly, abstinence was found consistently higher among employed individuals compared to those who were unemployed or street based. Furthermore, longer abstinence duration was found among those individuals who had a monthly income of 10,000 PKR-30,000 PKR. Statistically significant associations were not found between abstinence from drugs and gender, age, duration of rehab, duration of IV drug use and marital status.

**Table II:** Abstinence from drugs.

	<3 Months - n(%)	3-6 Months - n(%)	7-10 Month - n(%)	>10 Months - n(%)	Total - n(%)	P-value
Duration in rehabilitation/ Days^	45(38.5-60)	60(40-60)	52(40-60)	40(40-60)	45(40-60)	0.195^¥^
Duration of IV drug abuse/Days^	912(60-2555)	1277(365-1825)	1460(730-2920)	912.5(265-2281.25)	1095(365-2007.5)	0.801^¥^
Age/ Years^	29(27.25-32.75)	32(27-35)	31(27-39.75)	31(26-35)	31(27-36)	0.079^¥^
Back to work^	40(15.5-56.25)	60(40-60)	60(40-60)	45(40-60)	45 (35-65)	0.065^¥^
**Gender**						
Male	36(97.3)a	102(99.0)a	37(97.4)a	32(100)a	207(98.6)	0.514^€^
Transgender/MSM	1(2.7)a	1(1.0)a	1(2.5)a	0	3(1.4)	
**Marital Status**						
Single	19(52.8)a	54(52.4)a	13(34.2)a	13(40.6)a	99(47.4)	0.357^€^
Divorced	0	4(3.9)a	2(5.3)a	0	6(2.9)	
Married	14(38.9)a,b	40(38.8)b	22(57.9)a	17(53.1)a,b	93(44.5)	
Separated	3(8.3)a	5(4.9)a	1(2.6)a	2(6.3)a	11(5.3)	
**Work status/Back to work**						
Yes	15(42.9)a	48(46.6)a	24(66.7)b	29(93.5)c	116(56.6)	<0.0001 ^Τ^
No	20(57.1)a	55(53.4)a	12(33.3)b	2(6.5)c	89(43.4)	
**Mode of travel to the ARTC**						
Self	9(24.3)a	24(23.3)a	11(28.9)a	21(65.6)b	65(31.0)	<0.0001 ^Τ^
Family	5(13.5)a	7(6.8)a	4(10.5)a	4(12.5)a	20(9.5)	
NGO	23(62.2)a	72(69.9)a	23(60.5)a	7(21.9)b	125(59.5)	
**Family Support**						
Parents	8(21.6)a	21(20.4)a	7(19.4)a	6(18.8)a	42(20.2)	0.033 ^λ^
Siblings	9(24.3)a	10(9.7)b	2(5.6)b	5(15.6)a,b	26(12.5)	
Spouse	4(10.8)a	9(8.7)a	4(11.1)a	6(18.8)a	23(11.1)	
Other	1(2.7)a,b	1(1.0)b	4(11.1)a	4(12.5)a	10(4.8)	
No support	15(40.5)a	62(60.2)b	19(52.8)a,b	11(34.4)a	107(51.4)	
**Employment Status**						
Employed	20(54.1)a,b	46(44.7)b	25(65.8)a	28(87.5)c	119(56.7)	<0.0001 ^λ^
Unemployed/Not supported lives with family	1(2.7)a	9(8.7)a	0a	2a	12(5.7)	
Unemployed supported by family	12(35.1)a	29(28.2)a	11(28.9)a	2(6.3)b	55(26.2)	
Street Based	3(8.1)a,b	19(18.4)b	2(5.3)a,b	0a	24(11.4)	
**Social Status (monthly Income)**						
<10000	5(13.5)a	13(12.6)a	6(15.8)a	1(3.1)a	25(11.9)	0.004 ^λ^
10000-30000	21(56.8)a,b	53(51.5)b	25(65.8)a,b	23(71.9)a	122(58.1)	
30000-60000	1(2.7)a	6(5.8)a	4(10.5)a,b	6(18.8)b	17(8.1)	
>60000	10(27)a	31(30.1)a	3(7.9)b	2(6.3)b	46(21.9)	

Τ Pearson’s chi square test,

€ Fisher Exact Test,

λ Likelihood Ratio test,

¥ Kruskul Wallis Test,

^ Median(IQR)

## DISCUSSION

According to this study, most of our study population belonged to the younger age group, with the ratio of single to married almost similar, with a low income, and lesser number had family support. According to our findings marital status, employment status, family support system and a reasonable income were the main reasons for adherence to ARV. Highest adherence to ARVs has been at >10 months of follow up (73%) in PWID who have rejoined their respective occupations. At 3 to 10 months of follow up we noticed adherence of > 65% ,similar to a meta-analysis ,done in low and high income countries, which showed a healthy association with employment.^13^ Furthermore, ARV adherence at > 10 months of follow up was actually very good (65.3%) among those employed and had an income of 10,000 - 30,000 PKR (which is middle income group). Interestingly in our study , those without any social support have been adherent to their medications much more (60%) at 3 to 10 months of follow up which is contrary to many studies which showcase social support as a big factor for adherence.^14^,^15^ Regularity with ARVs has been more amongst married people (53%) when followed up for more than 10 months. We also saw a positive relationship between marriage and adherence at less than three months of follow up (50%). A further elaboration on this would be the quality of the relationship as a study done by Johnson et al emphasized a harmonious commitment to fare well in the level of adherence as well as virological suppression.^16^

When it comes to abstinence from drugs, similar results were found in the middle income group (71.9%). Those abstinent from drugs > 10 months had a positive association with self-transport (65%) which probably shows their commitment to getting well. Those who went back to work also were abstinent from drugs (93.5%) which makes going back to work a major factor to stay off recreational drugs, similarly those who were employed were also more inclined towards drug abstinence (87.5%). Most people with no social support were abstinent for 3 to 10 months only which dipped after that duration in our cohort which may show a need for family support to prevent relapsing. In addition, abstinence from recreational drugs was related favorably with job status, family support, a good income and clinic visit support. Unlike other studies, abstinence had no significant association with being married in our study.^17^

This negates our initial hypothesis that getting patient rehabilitated in a rehabilitation center is beneficial for adherence to ARVs and establishes abstinence to recreational drugs. Prior studies on the same subject synchronized with our findings that the effect of isolated educational interventions was not durable, suggesting that long-term or repetitive interventions may be required to produce a lasting impact on adherence and lifestyle modification as well as abstinence from drugs.^11^ Goujard et al. resonated with our findings of a reasonable income leading to better adherence in HIV treatment, however their findings of an educational system leading to good medication adherence were in contrast to our^18^ findings (which were of short-term 4 to 8 weeks of rehabilitation and education), and suggests that long term and repetitive rehabilitation and educational interventions are needed for sustained adherence. ARV adherence is casual if the person is homeless and without family support as suggested by our study in sync with a Canadian study.^19^ Alcohol use and depression have been found to be a cause of lax attitude towards ART.^20^ High pill burden and dosing frequency are also well studied factors for non-adherence^21^ which our study was not looking into.

### Limitations of the study:

We did not look at the quality of the marital life, depression and anxiety, other medical conditions along with HIV and education level of the PWID which may also impact adherence to ARV and abstinence to drugs. Another aspect which needs to be looked at is why most of our PWID are men and not women which our study fails to delineate. The strength of the study lies in exploring associations of various factors apart from rehabilitation only, which gives us an outlook on other aspects which may affect both adherence and abstinence.

## CONCLUSION

Our study concludes that for sustained ARV adherence rehabilitation programs and educational interventions must take place at regular intervals throughout the life of the patient. Furthermore, at each clinic visit family support system must be encouraged and revisited throughout therapy. Also efforts must be taken to encourage employment when starting treatment. However more research is needed to understand how long educational and rehabilitation programs should continue and whether it should be clinic/organization based or community based, one on one or group sessions and how frequently to assess for psychological ailments.

### Authors Contribution

**AH**: conception of the idea, designing the study, literature search, data compilation, write up and reviewing the article and responsible for the accuracy and integrity of this manuscript.

**AR**: data analysis, reviewing the article, write up

**AS**: data collection, compilation, data cleaning and management, reviewing the article

**AJ**: data Analysis, reviewing the article
